# Primary prevention of maternal anaemia to prevent preterm delivery and other adverse outcomes (PANDA): a protocol for a double-blind placebo-controlled randomised trial of a daily iron supplement during pregnancy

**DOI:** 10.1136/bmjopen-2026-122579

**Published:** 2026-09-28

**Authors:** Simon J Stanworth, David Churchill, Catherine Bain, Cara Hudson, Rosie Brown, Eleanor Hounslea, James Griffiths, Stephanie Lax, Joanne Murray, Helen Spiby, Noemi Roy, Andrew Farmer, Chris Gale, Elise Crayton, Fabiana Lorencatto, Marian Knight, PANDA Collaborator Group

**Affiliations:** 1 NHS Blood and Transplant, Oxford, UK; 2 Oxford University Hospitals NHS Foundation Trust, Oxford, UK; 3 Radcliffe Department of Medicine, University of Oxford, Oxford, UK; 4 The Royal Wolverhampton Hospitals NHS Trust, New Cross Hospital, Wolverhampton, UK; 5 Research Institute in Healthcare Science, University of Wolverhampton, Wolverhampton, UK; 6 Clinical Trials Unit, NHS Blood and Transplant, Bristol, UK; 7 Nottingham Maternity Research Network, School of Health Sciences, University of Nottingham, Nottingham, UK; 8 School of Health Sciences, University of Nottingham, Nottingham, UK; 9 Nuffield Department of Primary Care Health Sciences, University of Oxford, Oxford, UK; 10 Neonatal Medicine, School of Public Health, Imperial College London, Chelsea and Westminster Hospital, London, UK; 11 Department of Clinical, Educational and Health Psychology, Centre for Behaviour Change, University College London, London, UK; 12 National Perinatal Epidemiology Unit, Nuffield Department of Population Health, University of Oxford, Oxford, UK

**Keywords:** Maternal medicine, Anaemia, Randomized Controlled Trial, NEONATOLOGY, Pregnancy

## Abstract

Iron deficiency anaemia (IDA) affects up to 30% of all pregnant women often causing symptoms including malaise and lethargy which can significantly impact daily life and well-being. Additionally, IDA is associated with serious complications, including an increased risk of bleeding, preterm birth, small for gestational age babies and rarely, but significantly, stillbirth. The most common current management approach reactively treats IDA once it is diagnosed, usually with an oral iron preparation such as ferrous sulphate or another ferrous salt. There have been calls to investigate a policy of prevention, to discover if outcomes can be improved. We therefore designed the primary prevention of maternal anaemia to prevent preterm delivery and other adverse outcomes trial, to assess whether the prevention of IDA during pregnancy, through routine iron supplementation from early pregnancy, will reduce the risks of significant adverse clinical outcomes for women and their babies.

**Design:**

Randomised, double-blind, placebo-controlled trial to evaluate the effectiveness of low-dose ferrous sulfate in the primary prevention of IDA, alongside an intervention to support medication adherence, with a parallel process evaluation to explore acceptability, fidelity and mechanisms of action.

**Participants:**

11 020 women in their first trimester of pregnancy, who are not anaemic and have no contraindication to oral iron, will be recruited from maternity units.

**Intervention:**

A tablet of ferrous sulfate 200 mg (65 mg of elemental iron) once daily compared with an identically matched placebo tablet, administered from recruitment in the first trimester (anytime ≤15 weeks+6 days gestation) through until 6 weeks post partum or when all of the tablets have been taken, whichever is sooner. Participants are supplied with a total of 240 tablets to cover the whole period of the study. An adherence intervention to support initiation and adherence to iron supplementation was also delivered.

**Outcomes:**

The primary outcome is a composite of preterm birth, small for gestational age, stillbirth and neonatal death. Secondary outcomes include development of maternal anaemia, post-partum haemorrhage, postnatal depression, breast feeding rates, quality of life scores, and in the neonate admissions to a higher level of care and early-onset infection. Long-term offspring neurodevelopmental follow-up will continue beyond this period.

This study will establish the clinical and cost-effectiveness of universal oral iron supplementation in pregnancy. (ISRCTN16425597)

**STRENGTHS AND LIMITATIONS OF THIS STUDY**

- A key strength is that this double-blind randomised placebo-controlled trial is powered to provide a definitive answer to the question, does preventing iron deficiency anaemia during pregnancy reduce the rate of stillbirth, neonatal death, preterm birth and small for gestational age.
- The large sample size means it is representative of the broad population of pregnant women and thus will be able to provide robust evidence for benefits and safety in a policy area of prevention of disease.
- The Interventional Medicinal Product (iron or placebo) prescribing is combined with a behavioural intervention to maximise adherence in the trial, which was designed to be easy to apply at scale if considered for wider practice beyond the completion of the study.
- Recruitment is limited to early pregnancy up to gestational age 15+6 weeks, and therefore excludes high-risk women who are later in registering their pregnancies with maternity care services.

## Background and rationale

 During pregnancy, there is an expanding demand for iron to support the increase in maternal red cell mass and the developing placenta and fetus.^1^ The daily demand for iron increases across gestation, reaching a maximum of around 7.5 mg to 8 mg by the third trimester.^2 3^ This represents approximately 10 times the daily requirement of a non-pregnant woman of reproductive age. The fetus acquires the majority (70%) of its iron needs in the second half of pregnancy.^4^ Consequently, a pregnant woman will often develop low iron stores as her pregnancy advances, and around a third of pregnant women develop iron-deficiency anaemia, most commonly at the time of maximal demand in the third trimester.^5 6^

The range of potential adverse outcomes associated with iron deficiency anaemia (IDA) are broad and include: preterm birth, low birth weight, postpartum haemorrhage (PPH), postnatal depression, stillbirth and neonatal death.^7–13^ Infants of women who suffered from anaemia during pregnancy are also more likely to be iron deficient in early life, and some studies suggest an association between maternal IDA and neurocognitive impairments in offspring.^14–16^

Given the limitations of current diets, it is highly unlikely that the increased demand for iron during pregnancy can be met through dietary changes alone.^17^ Consequently, a woman relies on her iron stores to supplement what she obtains through her diet during pregnancy. These iron stores may have been depleted due to heavy periods or prior pregnancies.

Anaemia during pregnancy has been recognised as a major public health issue for many years. The current mainstream approach to management of anaemia in the UK and many high-income countries is based on a ‘screen and treat strategy’,^18^ as described in guidelines.^19^ However, this approach can be best considered as ‘reactive’, and with high demands on maternity services, there are often delays initiating treatment with inadequate follow-up, making it difficult to monitor treatment response and ensure its effectiveness, or to investigate other causes of anaemia.^6^

An alternative strategy to the policy of screen and treat proposes that preventing IDA in pregnancy will be more effective for both the woman and her baby. A recent systematic review^13^ evaluated studies comparing daily and intermittent iron supplementation in pregnant women for prevention of maternal anaemia. The majority of the 26 included studies were conducted in resource poor country settings, and most studies were deemed at high-risk of bias. The majority used haemoglobin concentration as their endpoint rather than clinical improvement.

A robust and well-designed trial of prevention of iron-deficiency anaemia powered on clinical outcomes is required.^20^ This protocol describes a definitive randomised trial, which has been co-designed with service users and is powered to assess the effect on clinical outcomes of significance to women and babies. The trial design will also support a future evaluation of the impact of prevention of IDA on long-term infant neurodevelopment. The study will include a process evaluation study to investigate intervention fidelity, feasibility and acceptability. This manuscript conforms to Standard Protocol Items: Recommendations for Interventional Trials (SPIRIT) guidelines^21^ (see online supplemental).

The trial was developed as part of a programme of research to address the burden of IDA in pregnancy.^22^ Earlier work included interviews with healthcare professionals and women to develop a behaviour change theory-based^23^ intervention to aid medication adherence (workstream 1). A dose-finding pilot trial of three doses of oral iron was undertaken to compare the treatment and side effect profile (workstream 2).^24^ In brief, the aim was to test adherence, the incidence of side effects and change in haemoglobin in preventing anaemia, of three different regimens of oral iron; a daily dose, alternate daily and three times a week. The results were considered by an independent international panel of experts. There were few differences between the groups and, after consideration of the findings, it was recommended that the daily dosing regimen be taken forward as it had the most potential to be efficacious and was as equally safe as the other regimens. Programme activities are supported by an active programme of patient public involvement and engagement (see public and patient involvment section).^24^

## Objectives

### Primary objective

To evaluate the clinical and cost-effectiveness of a primary prevention strategy for IDA in pregnancy using an oral iron supplementation intervention.

### Secondary objectives

To conduct a process evaluation to assess the fidelity, feasibility and acceptability of iron supplementation and a medication adherence intervention. This will inform interpretation of the clinical trial results, intervention refinement and subsequent scalability for general implementation.

### Design

A double-blind, randomised controlled trial evaluating the effectiveness of a once-daily dose of ferrous sulfate (200 mg equivalent to 65 mg of elemental iron) versus a placebo. The interventional medicinal product (IMP) will be given from the first trimester of pregnancy (≤15 weeks + 6 days gestation or less, with a ten day window to start) until 6 weeks post birth, or until all of the tablets have been used, whichever is sooner. Both arms of the trial include the adherence intervention developed in the earlier workstreams.^24^

In parallel, a mixed-methods process evaluation will be conducted guided by the Medical Research Council (MRC) guidance for process evaluations of complex interventions,^25^ to assess the process outcomes of fidelity, feasibility and acceptability (details in the online supplemental appendix). Additional follow-up will continue for neurodevelopmental outcomes through data linkage.

The pragmatic multicentre design is consistent with current pathways of care for pregnant women in the UK.

### Trial setting

Participants will be recruited from a mixture of secondary and tertiary care maternity hospitals and community antenatal clinics in the UK during the first trimester of pregnancy. The aim is to recruit centres from a variety of settings, urban, rural, inner city, to reflect the range of sites where maternity care is delivered. It is also considered to seek international recruitment, but ensuring that maternity pathways, baseline anaemia rates and causes are similar in any settings to UK populations.

### Trial participant identification

A range of methods, developed and refined as part of the pilot trial, will be used to notify and identify women suitable for trial entry.^24^ Written media will be provided to the range of professionals who have contact with and care for pregnant women in a variety of formats. Digital media in the form of videos will be made available on a variety of platforms including public websites and social media. Maternity units’ websites and electronic patient records (EPRs) will also be used where available. Women attending the maternity unit for antenatal clinic or ultrasound scan appointments will also be approached face to face by local research teams. The teams can also make direct approaches via telephone or video calls.

### Recruitment and consenting

Participants will be recruited from maternity units, including hospitals and community antenatal clinics in the UK. They will be identified through routine antenatal care and informed about the trial as early as possible. The participant information sheet (PIS) will also be available in paper form, as well as electronically.

Eligibility will be assessed using first-trimester blood tests (≤15+6 weeks gestation) and medical records. A full list of the inclusion and exclusion criteria is shown in box 1. Recruitment strategies include written materials (pregnancy packs, invitation letters, posters), midwife referrals, digital notifications (EPR alerts, SMS, QR codes), public engagement (social media, trial website, patient and public involvement groups) and direct contact by research teams, with specific attention to the importance of full follow-up for participants.

Box 1 **Eligibility criteria**

**Eligibility criteria**

- Participant inclusion criteria

Women need to meet the following inclusion conditions:

- Non-anaemic pregnant women of all parities (haemoglobin concentration ≥110 g/L measured by the first trimester blood profile at booking or screening).
- A live fetus on a first-trimester ultrasound scan carried out for viability or dating.
- 15 weeks+6 days gestation or less at consent.
- Aged 18 and above.
- Able to give informed consent and willing to fulfil trial requirements.

- Participant exclusion criteria.

Women with the following conditions will be excluded:

- Known major haemoglobinopathies (women with haemoglobinopathy trait are still eligible).
- Anaemia of any type, defined by British Society for Haematology guidelines.^19^
- Haematological conditions that require ongoing treatment with either regular oral or intravenous iron or transfusions, for example, syndromes of bone marrow failure.
- Severe gastrointestinal disease, requiring ongoing treatment and potentially affecting tolerability of oral iron.
- Chronic renal failure (requiring replacement therapy).
- Allergies to iron.
- Multiple pregnancies, given the higher iron demands.
- Known inherited haemochromatosis.
- Recent red cell transfusion, within 30 days.

Eligibility confirmation will be performed by the principal investigator (PI) or a delegated team member before consent and documented in medical records. Participants may only enrol once and will not be re-randomised in subsequent pregnancies. The informed consent process includes educational videos on iron supplementation, trial procedures and testimonials, as developed and refined during and following the pilot trial.^24^ A QR code links to trial updates and adherence support materials. Participants will provide electronic consent (e-Consent), countersigned by the PI or a delegated professional. Consent will be documented in medical records. Participation is voluntary, and withdrawal will not affect standard pregnancy care.

### Public and patient involvement

Public and patient involvement has been integral to the whole programme. Two service users are included on the trial management group. The study protocol, along with the previous studies of the research programme, has been codesigned with user input at all stages.^24^ Users are central, given the importance and implications of the trial should it prove to have a positive outcome. They will continue to be involved in the analysis, write-up and dissemination of the results when the trial is complete.

### Outcome measures and end points

To maximise the efficiency of the trial and reduce the burden on participating sites, routine, accurate and reproducible data will be extracted at source from the maternity and neonatal information systems and used for the analysis of the effectiveness of the intervention. For example, the practice for measuring birth weight is to do this as soon as possible after birth, usually within 30 min, and no more than 1 hour, using calibrated scales. Likewise, gestational age is calculated from the crown rump length, measured at the dating scan between 9 and 14 weeks gestation. Non-routinely collected data items will be kept to a minimum. All sites will be provided with relevant database training.

### Primary outcome

The primary outcome is a composite assessing the proportion of pregnancies resulting in preterm birth (<37 completed weeks gestation), stillbirth (≥24 weeks gestation), neonatal death (up to 28 days post partum) or small for gestational age (SGA) at birth (<10th centile sex-specific weight for gestational age, defined by UK growth charts).^26^

### Secondary outcomes

Secondary outcomes have been chosen to enhance the understanding of the wider impact of iron supplementation on maternal and infant health, and the underlying mechanisms mediating these effects. This includes outcomes to assess the safety of the intervention. Assessment of adherence will be measured through the Medication Adherence Report Scale (MARS-5) questionnaire at 28 weeks of gestation and again at 6 weeks post partum.^27^

Also included are exploratory outcomes to inform future long-term follow-up of the infants. Women who develop anaemia present an opportunity to study the effect of treatment in the context of the controlled environment of a trial. The impact of their treatment on health-related quality of life (HRQoL) and other outcomes, including PPH and mode of birth, will also be investigated as exploratory outcomes. Box 2.

Box 2 **Secondary outcomes**

**Secondary outcomes**

Relating to the mother are as follows:

- Components of the composite primary outcome, proportion of women with:
  - Preterm birth (<37 completed weeks gestation).
  - Small for gestational age (<10th centile sex-specific weight for age).
  - Stillbirth (at 24 weeks gestation or above).
  - Neonatal death (up to 28 days).
- Proportion of women developing anaemia during pregnancy.
- Transitions in haemoglobin from recruitment to 28 weeks and birth.
- Proportion of women with primary postpartum haemorrhage.
- Proportion of women who require red cell transfusions prior to discharge but not more than 48 hours post birth.
- Proportion of women who receive an iron infusion prior to discharge and up to 6 weeks after birth.
- Proportion of women with an infection and/or sepsis before discharge and up to 6 weeks after birth.
- Proportion of women breastfeeding or providing breast milk for baby at discharge from maternity care and at 6 weeks after birth.
- Proportion of women with postpartum depression assessed at 6 weeks post birth.
- Median adherence to medication at 28 weeks and 6 weeks post birth, as measured using the MARS-5 score.
- Health-related quality of life using EuroQol 5-Dimension 5-Level (EQ-5D-5L) at baseline, 28 weeks and at 6 weeks post birth.
- Healthcare utilisation over the trial period.

Relating to the infant are as follows:

- Mean birthweight.
- Mean gestation at birth.
- Apgar score at 5 min.
- Proportion of infants treated for neonatal early-onset infection, defined as culture-positive, occurring within 72 hours or prior to discharge home, whichever occurs first.
- Admission to a neonatal unit.
- Healthcare utilisation, including hospital stay, and subsequent hospital readmissions, up to 6 weeks post birth.

Relating to the process evaluation are as follows:

- Intervention fidelity.
- Intervention acceptability.
- Intervention feasibility.

### Interventional medicinal product

The IMP will be provided in the form of identical tablets (ferrous sulfate or placebo) packaged into indistinguishable small kits which are then placed into dispensing boxes before distribution to sites in accordance with the randomisation sequence. They will be removed sequentially at the recruiting centres as a participant is recruited. Participants are provided with enough medication for the entire trial.

### Cointerventions

The pragmatic nature of the trial precludes the collection of dietary information. The aim is to recruit a study sample that reflects the maternity population in all its diversity. Therefore, diets are likely to vary according to ethnicity and socioeconomic status. The randomisation process will account for any potential bias from different dietary preferences across the different population subgroups. Women who elect to take over-the-counter (OTC) vitamin or other supplements that contain iron in small amounts will not be excluded, and data on this additional form of supplement use will be collected. The use of OTC medication should be balanced between the two arms of the trial through the randomisation process and this will be reported.

All other care delivered to participants will follow local guidelines and be delivered by maternity healthcare professionals as per standard care for all pregnant women. No other changes will be made to their pregnancy care plan.

### Adherence intervention

Adherence to the trial medication is encouraged in two main ways. Table 1 First, through the provision of the adherence intervention at recruitment, comprising information about anaemia and use of iron supplementation, suggestions of strategies to enhance adherence, use of action planning, prompts to adherence and problem solving, as well as support to self-monitor adherence to iron supplements. It is delivered in a variety of ways using written information, videos and midwife discussions. Second, through text messages—a further element of the adherence intervention—delivered weekly during the trial. This multifaceted intervention supports initiation and continued adherence to the trial medication, and involves a combination of midwife brief advice, paper-based tools, weekly text messages and website-hosted media to deliver behaviour change techniques to provide information about anaemia in pregnancy, iron supplementation, support action planning and problem solving, and deliver ongoing prompts and cues. The adherence intervention was then piloted in the dose-finding pilot trial of the PANDA programme.^24^

**Table 1:** The adherence intervention

Timing of delivery/week of gestation	Mode of delivery	Intervention component	Description of the intervention component
e-consent module at recruitment/approx. 9–15 weeks +6 days gestation or less	Website (via signposting short message service/ quick response, SMS/QR, codes)/at recruitment and/or randomisation and provision of medication appointment and in community	PANDA videos	Information videos embedded within e-consent module and on PANDA website to provide the following content: Information about the purpose of the trial Facts and myths about using iron in pregnancy and for prevention of anaemia Testimonials from women in WS2 on their experiences of taking iron and participating in the PANDA study
At recruitment and/or randomisation and provision of medication appointment and when in community/approx. 9–15 weeks +6 days gestation or less and beyond when in community	Website (via signposting SMS/QR codes)	PANDA website	When women attend the clinic at the point of recruitment/kit visit**, they will be given a QR code** to access the PANDA website which can be read while waiting for the appointment The website will include: Information about the purpose of the trial Information about anaemia and iron for prevention Problem-solving strategies to help women identify any challenges with adherence to iron and potential solutions they can try PANDA newsletters
At randomisation and provision of medication appointment/approx. 9–15 weeks +6 days gestation or less	Face-to-face	Verbal midwife support	Midwives will ask women 2 questions. Question 1, when discussing the trial prior to consent, will check women’s understanding of information provided about the purpose of the trial and use of iron during pregnancy. Question 2 at the point women are given their iron tablets, will ask women if they anticipate any challenges to taking iron as recommended. Midwives will use PANDA resources described below to identify solutions to the challenges.
At randomisation and provision of medication appointment and when in community/approx. 9–15 weeks +6 days gestation or less and beyond when in community	Paper materials provided through initial verbal communication Materials will also be available to view on the PANDA website	Paper materials	3 paper materials will be provided and briefly discussed: **Provision of a paper document** presenting a diagram of common challenges and potential solutions to adhering to iron regimen (also available on PANDA website). **Action plan** to be completed in writing between midwife and participant following discussion of iron regimen and any predicted challenges with taking. A **self-monitoring tick chart** presenting days of the week in columns and number of weeks in rows. Women will be asked to record each day they take their iron, These materials will be used independently by participants when in the community.
1 per week when in community for up to 31 weeks/throughout pregnancy when in community once enrolled into study	Text messages	Text messages	**Midwives will check that women opt in to receive text messages**. One weekly text message which will include a sequence of alternating messages covering topics such as: A reminder to take iron And either (a) signposting to website content or (b) an embedded Behaviour Change Technique that provides advice on how to help adherence to iron regimen, for example, “PANDA: Setting daily alarms on your phone can help you get into the habit of taking your iron tablets. See (insert URL) for more help and support.”
When in community quarterly/throughout pregnancy when in community once enrolled into study	Website (via signposting SMS) Email	PANDA newsletter	Quarterly newsletter providing women with updates on rates of recruitment, any emerging evidence about use of iron in pregnancy and if appropriate testimonials from midwives and women involved in the study (fully anonymised)

Text in bold indicates activities research midwives will be administering and non-bold text indicates activities that participants will carry out independently.

The adherence intervention.

The table presents the individual intervention components, the time they will be delivered and the mode of delivery.

Text in bold indicates activities research midwives will be administering, and non-bold text indicates activities that participants will carry out independently.

PANDA, primary prevention of maternal anaemia to prevent preterm delivery and other adverse outcomes; WS2, work stream 2 of the PANDA trial.

### The randomisation scheme

A randomisation list will be generated using permuted blocks of variable undisclosed size. Kit numbers will be assigned in consultation with the packaging and distribution company to maintain blinding. Simple randomisation with no stratification will be used. However, sites will be sent shippers containing equal numbers of iron and placebo kits, thereby controlling balance by site.

### Method of implementing the randomisation/allocation sequence

Participating sites will be supplied with sealed, individually numbered kits. Women will be randomised by allocating them the next sequentially numbered pack once eligibility is confirmed and informed consent obtained.

### Blinding

In this trial, neither the participants, site staff nor investigators will know which intervention a participant is receiving until the clinical trial is completed and the trial database is locked.

### Emergency unblinding

An emergency unblinding facility will be available 24 hours per day, 7 days a week, although emergency unblinding is considered highly unlikely. Clinicians requesting emergency unblinding must be satisfied that knowledge of the participant’s allocation (to either ferrous sulfate tablets or matched placebo) is necessary for urgent clinical management.

### Adherence intervention unblinding

For the acceptability interviews, to be held with a subgroup of participants towards the end of the study, unblinding of a subset of participants is required in order to identify who received the active treatment (further details below). Once a minimum of 400 participants have completed all other data collection, an independent statistician will randomly select a subgroup of 200 participants who received iron and pass on this information to the adherence intervention team for them to contact participants for interview. The adherence intervention team will not disclose to the rest of the study team which participants have been contacted. Data collection on these participants will have been completed, and they are unlikely to have maintained contact with their local study teams. Nevertheless, the participants will be asked not to disclose to which of the trial arms they have been allocated to their local research study teams. The selection process and interviews will be carefully timed to ensure they do not extend beyond the end point of the study.

### Development of anaemia

No additional blood sampling will be undertaken for the trial. Data on haematological indices will be taken from the routinely taken samples at first registration of the pregnancy in the first trimester and then at 28 weeks gestation. Data will also be collected on routine haematological indices at the time of making a diagnosis of anaemia, if that is outside of the routine screening time points. Women who develop anaemia during the trial will be offered treatment as per local guidelines. They will remain in the trial for data collection purposes (and contribute to the analysis) but will discontinue taking the trial IMP. After commencing iron treatment (most commonly oral iron initially using the medication available at local hospitals), their response to treatment will be assessed at 2–4 weeks follow-up, as per national guidelines.^18^

### Trial timeline

The timings of the data collection, and assessments made of adherence and quality of life, are provided in figure 1. Consent for future data linkage to routinely recorded clinical and educational data, for women and their offspring, will be sought from all trial participants at trial enrolment. Linkage to routinely recorded clinical data including Hospital Episodes Statistics, and routinely recorded education data including National Pupil Database, will be undertaken as a future piece of work beyond the trial timeline.

**Figure 1:**
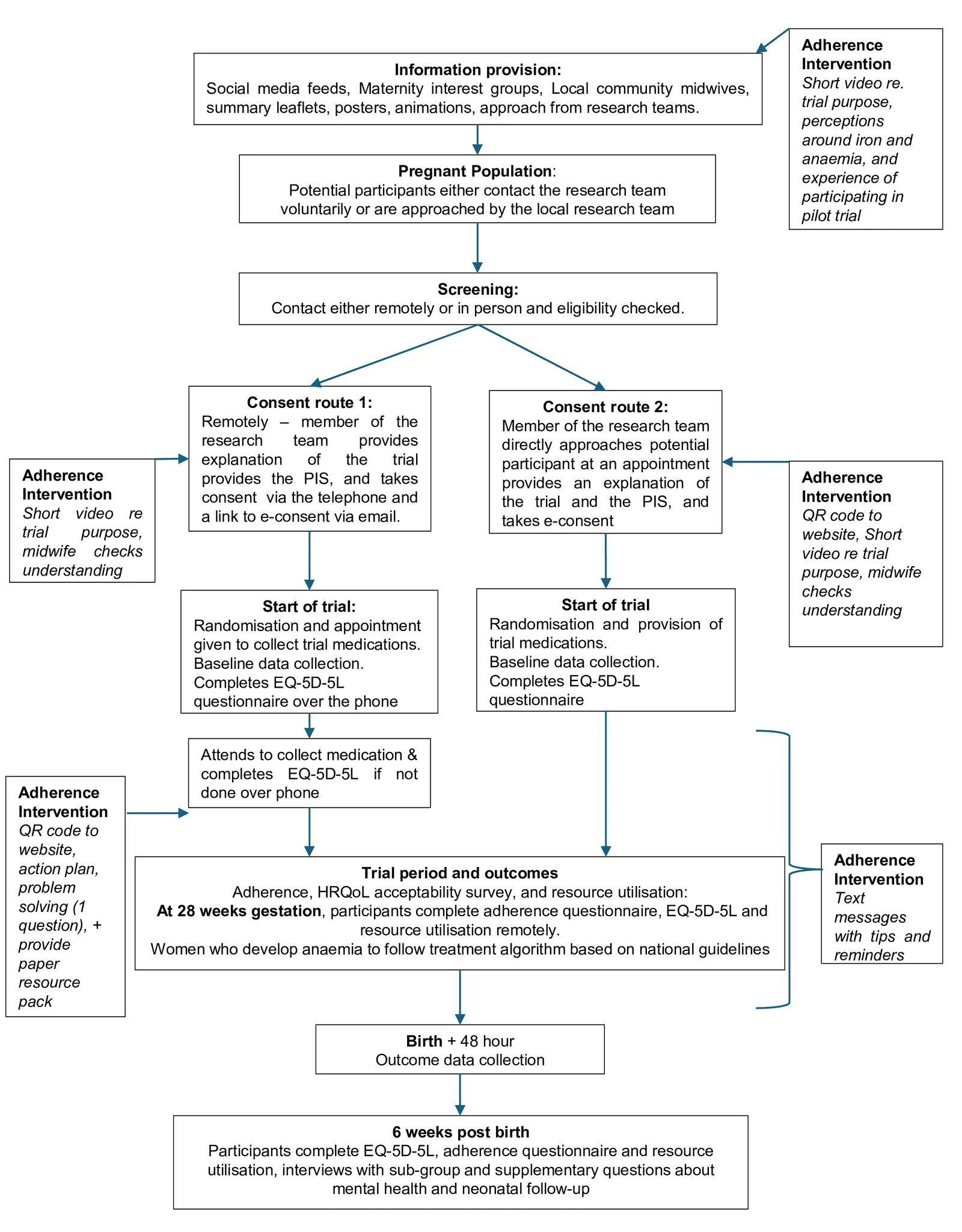
A figure to illustrate the timings of participant engagement and data collection. EQ-5D-5L, EuroQol 5-Dimension 5-Level; HRQoL, health-related quality of life; PIC, participant information sheet.

### Data handling and record keeping

Sites will enter participant data onto the trial database using OpenClinica. The OpenClinica database will be used for electronic data capture management and reporting on this trial. Training and instructions for completion of electronic Case Report Forms will be given to each site at site activation. Data sourced through linkage applications will be held securely in accordance with data security standards for NHS Blood and Transplant. A full site monitoring plan will be developed for the trial.

### Data linkage to routinely recorded data sources

Data to inform analysis of neonatal and maternal outcomes and health economic outcomes will be obtained through hospital Information Technology (IT) systems or linkage of trial data to routinely recorded clinical data held in national databases.

Data to inform neonatal admissions, deaths between birth and 6 weeks postnatal age which occur in neonatal care, and to inform neonatal exploratory outcomes will be collected via linkage to the National Neonatal Research Database (NNRD). Admissions and deaths for infants that occur outside neonatal care will be captured via hospital IT systems. Maternal admissions from entry point into the study until 6 weeks post partum of the study will be captured via the same method. Data to inform health economic evaluations will be collected via hospital IT systems also; however, consent will be obtained to allow linkage to NNRD, Office for National Statistics (ONS), Hospital Episode Statistics, Secure Anonymised Information Linkage Databank (Wales) and equivalents in other devolved nations, in the occurrence where the required study data mentioned above cannot be extracted by the original method.

## Statistics and data analysis

### Sample size calculation

The primary outcome is a composite measure of preterm birth, stillbirth, neonatal death and SGA. A study conducted in NHS hospitals in Wolverhampton and London involving 14 001 women^10^ found that 17.4% of pregnancies resulted in stillbirths, neonatal deaths, SGA births and preterm births. A Cochrane Review of pregnancy iron supplementation^28^ reported the relative risk of preterm birth for iron vs placebo as 0.82 (95% CI 0.58 to 1.14). Similar relative risks were observed for low birth weight and neonatal death (0.84 (95% CI 0.69 to 1.03)) and 0.91 (95% CI 0.71 to 1.18), respectively.

As some infants may experience multiple components of the composite outcome (eg, preterm babies are more likely to die in the neonatal period), the number of cases experiencing the composite outcome will not be additive. Therefore, to address whether prophylactic iron reduces the composite outcome rate, a conservatively relative risk of 0.85 was assumed, with a baseline rate of 15% and target rate of 12.75%. Using 90% power and a 5% significant level, the required sample size was calculated as 4959 participants in each group (9918 total).

Based on findings from other studies and antenatal trial data (Collaborative Low Dose Aspirin Study in Pregnancy Collaborative Group),^29 30^ a 10% drop-out was accounted for. Therefore, the final total number of participants required is 11 020. Data from earlier work further confirmed that a 10% drop out rate is appropriate; 23 participants withdrew, and two additional participants were lost to follow-up out of the 300 participants recruited in the previous dose finding trial.^24^

### Statistical analysis plan

#### Summary of baseline data and flow of patients

The characteristics of all randomised participants will be tabulated by arm of the trial to describe the cohort. A Consolidated Standards of Reporting Trials (CONSORT) diagram will be presented to show the flow of the participants through the trial.

#### Primary outcome analysis

For the main analysis of the primary outcome (and secondary maternal outcomes, unless otherwise specified), the analysis population will be intention-to-treat, with all participants in the arm to which they were randomised included. The primary outcome will be analysed using mixed logistic regression to allow for adjustment for centre as a random effect. The number needed to treat and summary statistics will also be presented.

The primary outcome, and all other analyses, will be based on an intention-to-treat basis, unless otherwise specified. As a sensitivity analysis, a per-protocol analysis of the primary outcome will be conducted which will exclude participants who withdrew from the trial, were randomised in error, were lost to follow-up, and experienced clinically significant protocol deviations.

### Secondary outcome analysis

#### Maternal and neonatal outcomes

Binary outcomes, including the individual components of the composite primary outcome (preterm birth, stillbirth, neonatal death, SGA), will all be analysed using a mixed logistic regression model adjusting for centre as a random effect. Descriptive statistics will also be presented, summarising the number and percentage with each of the outcomes, stratified by arm and overall.

The mean (SD) haemoglobin at 28 weeks and the adjusted mean change in haemoglobin between baseline and 28 weeks will be analysed using a mixed Normal model, with adjustment for centre as a random effect. Additionally, the model will adjust for the participant’s haemoglobin levels at baseline to account for individual variability. A similar approach will be taken with haemoglobin at delivery and its adjusted mean change between baseline and delivery. However, as haemoglobin at delivery is more likely to be reported in participants who develop anaemia, or experience preterm delivery, no formal statistical comparisons will be performed to compare between the two treatment arms.

Adherence with medication at 28 weeks and 6 weeks following delivery will be assessed using MARS-5 scores (0–25) and summarised descriptively. It is anticipated that these data will not be normally distributed; therefore, appropriate transformations will be applied to formally test for differences between the two treatment arms.

For neonatal outcomes, the analysis population will include only those pregnancies which resulted in a live birth. Similar analysis approaches to the maternal outcomes will be taken.

### Safety outcomes

Serious adverse events (SAEs) will be summarised separately for maternal and infant populations, stratified by treatment arm and overall. No formal statistical comparisons will be made, beyond those already included in the maternal and neonatal outcome analysis. Detailed listings of SAEs will also be produced.

### Exploratory outcome analysis (through data linkage or hospital IT systems)

For the neonatal exploratory outcomes, summary statistics will be presented by treatment arm and overall. Apart from late-onset infection, which will include all those with a live birth pregnancy outcome, the analysis populations used for each outcome will align with the National Neonatal Audit Programme (NNAP) and NNAP definitions for the outcomes will be used, where applicable.

For the exploratory outcomes related to the treatment of anaemia in women recruited to the trial, summary statistics will be presented for all outcomes, with no formal statistical testing conducted.

- HRQoL will be summarised using descriptive statistics. For those participants diagnosed around the 28-week visit, additional descriptive statistics will be used to summarise changes between EuroQol 5-Dimension 5-Level (EQ-5D-5L) scores at 28 weeks and the 2–4 weeks treatment timepoints.

Other outcomes, including PPH and mode of delivery, will be summarised in line with the descriptive statistics presented for the corresponding secondary outcomes, as outlined above.

### Subgroup analyses

The primary outcome analysis will be replicated in several subgroup analyses to explore differential treatment effects on outcomes. These are:

- Maternal age (<25 years vs 25–35 years vs >35 years).
- Ethnicity (white and ethnic minority groups).
- Measures of iron status at randomisation (eg, lower haemoglobin concentration or Mean Cell Volume or ferritin may indicate borderline/low iron stores and hence participants may respond better to oral iron supplementation, reducing the likelihood or experiencing the primary outcome).
- Time since last pregnancy (≤18 months vs >18 months between birth and the due date for the current pregnancy).
- Gestational age at randomisation (≤12 weeks vs >12 weeks).

### Interim analysis and criteria for the premature termination of the trial

An interim analysis will be performed to re-assess the sample size calculation after the primary outcome data have been reported for at least 1500 participants. This calculation will estimate the overall primary outcome rate (not calculated by arm) and the dropout rate, with all other assumptions unchanged. The Data Monitoring Committee, who advise the Programme Steering Committee (PSC), will review the interim analysis as well as accruing unblinded trial data, in particular safety data, at least twice a year as part of their overall assessment of the trial. The ultimate decision on continuation of the study lies with the PSC.

### Procedure(s) to account for missing or spurious data

Data completeness will be regularly reviewed by the data and trial managers as the trial progresses and any missing data will be requested. Any outstanding missing data at the end of the trial will be assumed to be missing at random and will be summarised accordingly. For the main analysis, missing data will not be imputed for primary and secondary outcome measures, and cases with the missing data will be excluded from the relevant analyses.

To explore if missing values have an undue impact on the primary outcome result, a sensitivity analysis using multiple imputation will be performed if more than 10% of the participants in the intention to treat analysis have missing primary outcome data.

However, as the primary outcome is identifiable through routine data collection at delivery, it is anticipated that missing data due to loss to follow-up will be minimal.

### Process evaluation

An integrated process evaluation will explore the extent of implementation and mechanisms of change that may contribute to interpretation of observed results from the trial. We will explore fidelity of the adherence intervention (ie, whether the intervention was delivered and enacted as planned) delivery by midwives and engagement by participating women. We will also explore intervention acceptability and feasibility. Further details are found in the online supplemental appendix.

### Economic evaluation

The objective of the economic evaluation is to determine whether the additional benefits of 200 mg ferrous sulfate compared with a matched placebo justify the additional costs needed to deliver the interventions within an NHS setting. An NHS perspective and a time horizon of 6 weeks post partum will be employed. The economic evaluation will assess whether the use of ferrous sulfate supplementation, as a preventive measure, translates into differences between groups in costs or outcomes driven by changes in any of the individual components of the composite outcome (preterm birth, stillbirth, neonatal death and SGA) or secondary outcomes (eg, maternal mental health).

The economic evaluation will be presented using two different strategies. The primary strategy will be a cost–consequence analysis, where maternal and infant costs and outcomes will be presented in a disaggregated manner over the trial period. A second strategy will be a costutility analysis, assessing the results from the woman’s perspective using quality-adjusted life-years (QALYs) over the trial period.

We will employ unit costs from national catalogues and the literature to estimate costs in each arm of the trial. Maternal QALYs up to 6 week post partum in each trial arm will be estimated as the area under the mother-specific utility profile generated using utility values from the EQ-5D-5L instrument. The currently National Institute for Health and Care Excellence (NICE)-recommended crosswalk will be used to derive utility values, and these will be connected using a straight-line relationship between follow-up points.

Uncertainty around cost-effectiveness results will be presented using parametric and non-parametric CIs for the incremental cost-effectiveness ratio (if appropriate) and net-benefits. For both cost-consequence and cost–utility analysis, mean differences and associated 95% CI between alternatives will be estimated using a normal mixed-effect model with centre as a random effect and adjusted (if necessary) for potential imbalances at baseline. Missing data in costs and uses will be imputed if necessary, using multiple imputation approaches.

### Ethical considerations

The trial protocol and all patient-facing materials were reviewed by the North of Scotland Research Ethics Committee (REC reference number 23/NS/0123; favourable opinion granted on 17 November 2023). Any substantial amendments to the protocol or patient-facing materials will be referred back to the committee for further consideration and approval.

### Dissemination of findings

At the conclusion of the trial, a final report will be prepared and submitted for publication in a peer-reviewed medical journal. The main trial results will be presented at relevant conferences, and all main trial publications will be made available through the trial website. Participants will have access to the reports through the website. Relevant ‘patient support groups’ aligned with the primary outcomes will also be made aware of the findings. Finally, the report will be available to guideline writing bodies who produce guidance on the topic for professionals and the public.

## Discussion

Iron is an important micronutrient in pregnancy that sustains the health of the woman and developing baby. However, gestational IDA is very common, affecting up to 30% of women. If IDA is inadequately treated, an all too often common problem, there may be increased risks for several important obstetric outcomes for the baby, including preterm birth, SGA and stillbirth. In addition, for the woman, it causes fatigue, malaise, poor exercise tolerance and predisposes to PPH and postnatal depression. Evidence is also emerging, although not proven, that maternal IDA detrimentally affects the neurocognitive development of babies, by affecting myelination, monoamine metabolism and epigenetic imprinting.^2 13 14^ These effects might contribute to the observed association between iron deficiency and long-term childhood neurological and psychiatric morbidities, including poorer fine motor skills, language abilities, cognitive functions and memory.^2 13 14^

Oral and intravenous iron treatments present challenges. Oral iron is often linked with gastrointestinal side effects, resulting in poor adherence or modification of intake, such as taking iron with food, both of which reduce overall iron intake and/or absorption.^2^ In turn, oral iron may detrimentally alter the gut microbiome especially in women who are iron replete, and thus lead to more serious infective episodes.^31^ Intravenous iron is increasingly being considered as an alternative but has higher costs, has specific risks (such as hypersensitivity reactions and skin staining) and requires additional resources for administration. There may be risks associated with iron administration in women who are potentially iron replete. Studies have linked iron in these situations to a higher risk of infections, gestational diabetes and pregnancy-induced hypertension, although the evidence is relatively weak.^32–34^

Women vary in the level of their iron stores at the commencement of a pregnancy. It is known that by the third trimester of pregnancy the daily requirement for iron increases 10-fold.^2 3^ This cannot be met through dietary changes and pregnant women rely on their iron stores to meet this need, exposing those with low stores to the development of anaemia.^35 36^ The challenges associated with the timely diagnosis and effective treatment of IDA in pregnancy have led to some recommending the prescribing of oral iron to women who they consider to be iron deficient, even though they are not anaemic. However, there is no agreement on which biomarkers or which thresholds (eg, ferritin) to use in pregnant women and thus the definition of what constitutes iron deficiency in pregnancy.^37^ The evidence for an association between ID and clinical outcomes fails to show statistical significance, unlike IDA where many studies and systematic reviews show an association with clinically worrying outcomes such as stillbirth, preterm birth and low birth weight.^38–41^ Therefore, producing a clear evidential basis for iron supplementation in all pregnant women requires a large trial of prevention of IDA to demonstrate if there is a genuine improvement in obstetric outcomes.

While an association does not confirm causality, the weight of research evidence linking IDA to adverse pregnancy outcomes appears strong. The associations remain when other confounding factors, such as socioeconomic status and smoking, among many others, are accounted for in the analyses. Also, using directed acyclic path analysis, we have shown that low haemoglobin levels contribute to the risk of stillbirth through different mechanistic pathways, although the exact nature of these mechanistic influences is yet to be defined.^11^ Overall, it is likely that there is an influence from IDA on obstetric outcomes, and is worthy of research investigations through the conduct of randomised trials.

Both oral and intravenous iron are not without side effects and safety concerns.^42–45^ Any intervention trialled with the potential to change policy at the population level must be shown to be safe before implementation. This is a focus of the PANDA programme as a whole and has been a stated part of the earlier workstreams, where we investigated gastrointestinal and other side effects resulting from oral iron. The side effect profiles did not show any differences between the three dosage schedules in our pilot trial. In fact, many of the symptoms associated with side effects of oral iron were already present before the iron was started.^24^ In the main trial, data are being collected on all the previously reported potential complications associated with oral iron, pre-eclampsia, gestational diabetes and infections in both the mother and neonate. They will be reported in the final analysis and a balanced risk/benefit conclusion reached on whether a universal policy of iron supplementation is not only effective but also safe.

This protocol describes an ongoing randomised trial aimed at evaluating the clinical-effectiveness and cost-effectiveness of universal oral iron supplementation in combination with an adherence intervention to prevent IDA during pregnancy. Recruitment is occurring across the UK, with plans for international sites to join. The trial design was informed by earlier behavioural work and a dose-finding study to identify an optimal iron dose.^24^ The trial opened on 23 October 2024, and the first participant was recruited on 29 October 2024.

A strength of the study is the blinded nature and the sample size, powered to answer clinically relevant outcomes of importance to the woman. Limitations reflect the pragmatic nature of the study which precludes detailed analysis of adherence and other outcomes, and the use of a neonatal composite primary outcome which combines death with preterm birth. It is a risk that recruitment may not reflect the sociodemographic make-up of the whole population. To monitor this potential limitation, each site will keep screening logs of women approached and declined to participate and women who were approached and agreed. The trial also includes a large number of participating sites that should cover a wide demographic.

Providing the adherence support to all participants, regardless of the arm of the trial, isolates the effect of oral iron. It is possible that any beneficial effect found may be diminished when generalising the process to the whole population because the behavioural support would not be available in full. This is a possibility, but it has been limited in two ways. First, the adherence intervention was codesigned with midwives, maternity care workers and pregnant women, and great care was taken to ensure that it was deliverable under normal practice conditions. Second, all trial materials will be made universally available at the end of the trial should there be a positive benefit from iron. Nevertheless, the text messaging reminders are not likely to continue and so there may be some residual loss of benefit, although this can be counteracted by the adoption of universal evidence-based advice by professionals providing care as has been adopted with folic acid supplementation and smoking cessation interventions.

We are not aware of any other large, randomised placebo-controlled trial in pregnancy combining a medical intervention, supplementary iron, with an adherence intervention. The two aspects together will give us the best chance of answering the research question on the role of oral iron supplementation to prevent anaemia. The study recognises that iron may have risks and increase the risk of infections, gestational diabetes and pre-eclampsia^12 36 46^; we are collecting data on all three of these potential clinical complications. It is expected that the characteristics of the sample population of pregnant women will closely mirror those of the general pregnant population. This is important considering that the trial’s findings could have implications for what is the implementation of a large-scale public health intervention.

## Supplementary material

10.1136/bmjopen-2026-122579 online supplemental file 1

## References

[1] Costantine MM (2014). Physiologic and pharmacokinetic changes in pregnancy. Front Pharmacol.

[2] Alwan NA, Hamamy H (2015). Maternal Iron Status in Pregnancy and Long-Term Health Outcomes in the Offspring. J Pediatr Genet.

[3] Andrews NC (1999). Disorders of iron metabolism. N Engl J Med.

[4] WIDDOWSON EM, SPRAY CM (1951). Chemical development in utero. Arch Dis Child.

[5] Barroso F, Allard S, Kahan BC (2011). Prevalence of maternal anaemia and its predictors: a multi-centre study. Eur J Obstet Gynecol Reprod Biol.

[6] Churchill D, Ali H, Moussa M (2022). Maternal iron deficiency anaemia in pregnancy: Lessons from a national audit. Br J Haematol.

[7] PRITCHARD JA (1965). CHANGES IN THE BLOOD VOLUME DURING PREGNANCY AND DELIVERY. Anesthesiology.

[8] Cao C, O’Brien KO (2013). Pregnancy and iron homeostasis: an update. Nutr Rev.

[9] Young MF, Oaks BM, Tandon S (2019). Maternal haemoglobin concentrations across pregnancy and maternal and child health: a systematic review and meta-analysis. Ann N Y Acad Sci.

[10] Nair M, Churchill D, Robinson S (2017). Association between maternal haemoglobin and stillbirth: a cohort study among a multi-ethnic population in England. Br J Haematol.

[11] Nair M, Knight M, Robinson S (2018). Pathways of association between maternal haemoglobin and stillbirth: path-analysis of maternity data from two hospitals in England. BMJ Open.

[12] Georgieff MK (2020). Iron deficiency in pregnancy. Am J Obstet Gynecol.

[13] Quezada-Pinedo HG, Cassel F, Duijts L (2021). Maternal Iron Status in Pregnancy and Child Health Outcomes after Birth: A Systematic Review and Meta-Analysis. Nutrients.

[14] Lynch S, Pfeiffer CM, Georgieff MK (2018). Biomarkers of Nutrition for Development (BOND)-Iron Review. J Nutr.

[15] Bastian TW, von Hohenberg WC, Mickelson DJ (2016). Iron Deficiency Impairs Developing Hippocampal Neuron Gene Expression, Energy Metabolism, and Dendrite Complexity. Dev Neurosci.

[16] Toolan M, Barnard K, Lynch M (2022). A systematic review and narrative synthesis of antenatal interventions to improve maternal and neonatal health in Nepal. *AJOG Glob Rep*.

[17] Getu S, Shiferaw E, Melku M (2020). Neonatal Iron: Factors Influencing its Level and Associated Complications - a Review Article. Clin Lab.

[18] Rahman MM, Abe SK, Rahman MS (2016). Maternal anaemia and risk of adverse birth and health outcomes in low- and middle-income countries: systematic review and meta-analysis. Am J Clin Nutr.

[19] Pavord S, Daru J, Prasannan N (2020). UK guidelines on the management of iron deficiency in pregnancy. Br J Haematol.

[20] Dewey KG, Oaks BM (2017). U-shaped curve for risk associated with maternal haemoglobin, iron status, or iron supplementation. Am J Clin Nutr.

[21] Hróbjartsson A, Boutron I, Hopewell S (2025). SPIRIT 2025 explanation and elaboration: updated guideline for protocols of randomised trials. BMJ.

[22] NIHR The ‘panda’ research programme: primary prevention of maternal anaemia to avoid preterm delivery and other adverse outcomes. https://www.fundingawards.nihr.ac.uk/award/NIHR200869.

[23] Michie S, van Stralen MM, West R (2011). The behaviour change wheel: A new method for characterising and designing behaviour change interventions. Implementation Sci.

[24] Stanworth SJ, Churchill D, Sweity S (2024). The impact of different doses of oral iron supplementation during pregnancy: a pilot randomised trial. Blood Adv.

[25] Moore GF, Audrey S, Barker M (2015). Process evaluation of complex interventions: Medical Research Council guidance. BMJ.

[26] Webbe J, Sinha I, Gale C (2018). Core Outcome Sets. Arch Dis Child Educ Pract Ed.

[27] Chan AHY, Horne R, Hankins M (2020). The Medication Adherence Report Scale: A measurement tool for eliciting patients’ reports of nonadherence. Br J Clin Pharmacol.

[28] Peña-Rosas JP, De-Regil LM, Garcia-Casal MN (2015). Daily oral iron supplementation during pregnancy. Cochrane Database Syst Rev.

[29] Collaborative Low-dose Aspirin Study in Pregnancy (1994). CLASP: a randomised trial of low-dose aspirin for the prevention and treatment of pre-eclampsia among 9364 pregnant women. The Lancet.

[30] Poston L, Briley AL, Seed PT (2006). Vitamin C and vitamin E in pregnant women at risk for pre-eclampsia (VIP trial): randomised placebo-controlled trial. Lancet.

[31] O’Toole F, Sheane R, Reynaud N (2024). Screening and treatment of iron deficiency anaemia in pregnancy: A review and appraisal of current international guidelines. Int J Gynaecol Obstet.

[32] Scanlon KS, Yip R, Schieve LA (2000). High and low haemoglobin levels during pregnancy: differential risks for preterm birth and small for gestational age. Obstet Gynecol.

[33] Shi H, Chen L, Wang Y (2022). Severity of Anaemia During Pregnancy and Adverse Maternal and Fetal Outcomes. *JAMA Netw Open*.

[34] Steer P, Alam MA, Wadsworth J (1995). Relation between maternal haemoglobin concentration and birth weight in different ethnic groups. BMJ.

[35] Zych-Krekora K, Sylwestrzak O, Krekora M (2025). The Critical Role of Iron in Pregnancy, Puerperium, and Fetal Development. J Clin Med.

[36] Malinowski AK, Murji A (2021). Iron deficiency and iron deficiency anaemia in pregnancy. CMAJ.

[37] Lawani LO, Naimark DM, Ray JG (2026). Iron deficiency with and without anaemia in pregnancy: A scoping review of prevalence and perinatal impact. Int J Gynaecol Obstet.

[38] Haider BA, Olofin I, Wang M (2013). Anaemia, prenatal iron use, and risk of adverse pregnancy outcomes: systematic review and meta-analysis. BMJ.

[39] Benson AE, Lo JO, Caughey AB (2024). Iron Deficiency and Iron Deficiency Anaemia During Pregnancy-Opportunities to Optimize Perinatal Health and Health Equity. *JAMA Netw Open*.

[40] Daru J, Colman K, Stanworth SJ (2017). Serum ferritin as an indicator of iron status: what do we need to know?. Am J Clin Nutr.

[41] Daru J, Allotey J, Peña-Rosas JP (2017). Serum ferritin thresholds for the diagnosis of iron deficiency in pregnancy: a systematic review. Transfus Med.

[42] Benson CS, Shah A, Frise MC (2021). Iron deficiency anaemia in pregnancy: A contemporary review. Obstet Med.

[43] Kortman GAM, Raffatellu M, Swinkels DW (2014). Nutritional iron turned inside out: intestinal stress from a gut microbial perspective. FEMS Microbiol Rev.

[44] Kortman GAM, Roelofs RWHM, Swinkels DW (2014). Iron-induced virulence of Salmonella enterica serovar typhimurium at the intestinal epithelial interface can be suppressed by carvacrol. Antimicrob Agents Chemother.

[45] Tolkien Z, Stecher L, Mander AP (2015). Ferrous sulfate supplementation causes significant gastrointestinal side-effects in adults: a systematic review and meta-analysis. PLoS One.

[46] Obianeli C, Afifi K, Stanworth S (2024). Iron Deficiency Anaemia in Pregnancy: A Narrative Review from a Clinical Perspective. Diagnostics (Basel).

