# Supplementary material for: Primary prevention of maternal anaemia to prevent preterm delivery and other adverse outcomes (PANDA): a protocol for a double-blind placebo-controlled randomised trial of a daily iron supplement during pregnancy
Source: BMJ Open. 2026 Sep 28;16(9):e122579. doi: 10.1136/bmjopen-2026-122579 (PMC13629978; doi:10.1136/bmjopen-2026-122579)
Supplement: Supplementary file 1 [file bmjopen-2026-122579.reviewer_comments.pdf]

PEER REVIEW HISTORY

BMJ Open publishes all reviews undertaken for accepted manuscripts. Reviewers are asked to complete a checklist review form and are provided with free text boxes to elaborate on their assessment. These free text comments are reproduced below.

ARTICLE DETAILS

Title (Provisional)

Primary prevention of maternal anaemia to prevent preterm delivery and other adverse outcomes (PANDA): a protocol for a double-blind placebo controlled randomised trial of a daily iron supplement during pregnancy

Authors

Stanworth, Simon J; Churchill, David; Bain, Catherine; Hudson, Cara; Brown, Rosie; Hounslea, Eleanor; Griffiths, James; Lax, Stephanie; Murray, Joanne; Spiby, Helen; Roy, Noemi; Farmer, Andrew; Gale, Chris; Crayton, Elise; Lorencatto, Fabiana; Knight, Marian; Collaborator Group, PANDA

VERSION 1 - REVIEW

Reviewer **1**

Name **Muñoz, Manuel**

Affiliation **School of Medicine, University of Málaga, Perioperative Transfusion Medicine**

Date **10-Jun-2026**

COI **None**

The author should be congratulated for the design and conduction of this RTC of overhelminn importance. The study results will most probably end the discussion around prophylactic iron supplementation during pregnancy.

Just some minor comments

Abstract.

Please, revise this sentence "Secondary neonatal short-term outcomes include mean birthweight, gestation at birth,

early onset infection rates, medication adherence, and intervention feasibility, acceptability and fidelity"

Methods.

Could you consider evaluating the effects of concomitant iron-rich diet? This could be relevant for the placebo arm, especially for those women also intaking OTC supplements containing iron.

As the newborn experiences a significant weight loss during the first 72 hours after birth, on my opinion, the timing for obtaining the birth .

weight should be clearly specified.

Have you considered the assessment of the incidence of non-anaemic iron deficiency, not was that of anaemia?

Wich is the estimated time for the RCT completion?

Reviewer **2**

Name **Saha, Lekha**

Affiliation **Department of Pharmacology, Post Graduate Institute of Medical Education & Research (PGIMER), Chandigarh 160012, India.**

Date **12-Jun-2026**

COI **None**

Major Concerns

Overstatement of the Causal Relationship Between Anaemia and Adverse Outcomes

Several statements imply causality between maternal anaemia and adverse pregnancy outcomes. For example: “The range of potential adverse outcomes associated with IDA include preterm birth, low birth weight, postpartum haemorrhage, stillbirth and neonatal death. “Although associations are well documented, causality remains incompletely established because: Anaemia may be a marker of underlying socioeconomic disadvantage, nutritional deficiencies frequently coexist and chronic infection and inflammation may contribute.

Inadequate Discussion of Potential Risks of Universal Supplementation

The rationale focuses almost exclusively on benefits. Potential risks are largely ignored, including: Gastrointestinal adverse effects , Reduced adherence , Iron overload in iron-replete women Alterations in gut microbiota and Oxidative stress. A balanced protocol should discuss both anticipated benefits and potential harms.

Lack of Consideration of Baseline Iron Status Heterogeneity

The protocol proposes supplementation in women who are non-anaemic but does not adequately discuss heterogeneity within this population. Participants may include: Women with depleted iron stores , Women with iron deficiency without anaemia and Fully iron-replete women. These groups may respond differently to supplementation. Potential Consequence may be treatment effects may be diluted if substantial numbers of iron-replete women are included.

Justification for Dose Selection Requires Further Detail

The protocol states that a pilot dose-finding study identified 65 mg elemental iron daily as the preferred dose. However, insufficient detail is provided regarding: Comparative efficacy of alternative doses , Side-effect profiles and Reasons for selecting the final dose

International Recruitment Creates Potential Heterogeneity

The protocol states: "It is also planned to seek international recruitment." This introduces several concerns: Differences in baseline anaemia prevalence, Variations in dietary iron intake , Different maternity care pathways and Different supplementation policies .

Limited Discussion of Long-Term Neurodevelopmental Outcomes

The protocol suggests future neurodevelopmental follow-up but states that this is dependent on funding. This creates uncertainty regarding: Feasibility , Outcome definitions and Assessment methods.

Potential Design Limitations

Reliance on Behavioural Adherence Support: Both study arms receive the adherence intervention. While this isolates the effect of iron supplementation, it may reduce generalizability to healthcare systems where such behavioural support is unavailable.

Recruitment Bias: Women willing to participate in a long-term supplementation trial may: Be more health conscious, Have higher health literacy and Demonstrate better medication adherence than the general pregnant population. This may limit external validity.

Revisions Required

1. Provide a more balanced discussion of potential risks and benefits of universal iron supplementation.
2. Clarify management of baseline iron-status heterogeneity.
3. Expand justification for the selected iron dose.
4. Clarify implications of planned international recruitment.
5. Avoid implying causality where evidence is primarily observational.
6. Provide greater detail regarding planned neurodevelopmental follow-up.

Recommendation: Minor to Moderate Revision

The protocol addresses a significant evidence gap in maternal health and is methodologically robust. However, clarification of several conceptual and methodological issues would improve scientific balance, transparency, and interpretability before publication.

**Reviewer**	**3**
**Name**	**Chipperfield, Kate**
**Affiliation**	**BC Children's Hospital Research Institute, Hematopathology**
**Date**	**13-Jun-2026**
**COI**	**None**

Thank you for the opportunity to review this paper.

Overall, the paper is organized and thoughtfully written. The trial protocol design is clear, and the results will be valuable to patients and practitioners. Note is made of significant background work contributing to this protocol's strength. Use of combined neonatal outcomes (rather than maternal hemoglobin), and addition of medication adherence strategy are interesting. I recommend the paper for publication.

Suggestions for improvement are noted here:

1. The document should have final editorial review for syntax. (Lines 65, 122, 180, 536-540, etc)

2. Include a statement indicating that the trial protocol and all patient-facing materials (advertisement, consent, adherence strategy interventions) have been reviewed by ethics board. I was unable to locate in body of paper; apologies if this is my error. Also, will materials and consent be available in languages other than English?

3. Anticipated start date for enrolment and projected rate of enrolment would be of interest.

4. Under "recruitment and consenting", an explicit statement that table 1 lists the inclusion and exclusion criteria would be helpful. (Line 210-11)

5. Per secondary outcome analysis and headings of "Development of Anemia" and Trial Timeline, it is unclear if blood sampling will be undertaken/ required by protocol, or is Hemoglobin assessment reliant on existing care guidelines or practitioners' clinical judgement? This is important to clarify since development of anemia and delta of hemoglobin are secondary outcomes. Also, if international centers are participating, their local care guidelines for hemoglobin assessment may differ. Will this be addressed?

6. The study targets and recruits only those women who seek early access to pregnancy care. This is understandable for this pragmatic trial. Authors could consider acknowledging in limitations that this trial excludes some women at risk of anemia and negative outcomes like SGA, preterm delivery or stillbirth.

VERSION 1 - AUTHOR RESPONSE

Reviewer 1

1. Abstract.
Please, revise this sentence "Secondary neonatal short-term outcomes include mean birthweight, gestation at birth, early onset infection rates, medication adherence, and intervention feasibility, acceptability and fidelity"

We have clarified the section and reworded it to bring more clarity to: "Secondary outcomes include development of maternal anaemia, post-partum haemorrhage, postnatal depression, breast feeding rates, quality of life scores and in the neonate admissions to a higher level of care, and early onset infection."

2. Methods.
Could you consider evaluating the effects of concomitant iron-rich diet? This could be relevant for the placebo arm, especially for those women also intaking OTC supplements containing iron.

We have clarified this on page 7 section Co-interventions by adding the sentences. We considered options for collecting data on diet, but recognized this is very challenging
"The pragmatic nature of the trial precludes the collection of dietary information. The aim is to recruit a study sample that reflects the maternity population in all its diversity. Therefore, diets are likely to vary according to ethnicity and socioeconomic status. The randomisation process will account for any potential bias from different dietary preferences across the different population sub-groups."
"The use of over the counter (OTC) medication should be balanced between the two arms of the trial through the randomisation process and this will be reported."

3. As the newborn experiences a significant weight loss during the first 72 hours after birth, on my opinion, the timing for obtaining the birth weight should be clearly specified. We have clarified the approach to data collection by adding the following under the heading Outcome Measures and End Points. This addresses the question relating to birth weight:
"We have clarified our approach to data collection and addressed the matter of birth weight measurement by adding the following to the section Outcome measures and End points:

To maximise the efficiency of the trial and reduce the burden on participating sites, routine, accurate and reproducible data will be extracted at source from the maternity and neonatal information systems and used for the analysis of the effectiveness of the intervention. For example, the practice for measuring birth weight is to do this as soon as possible after birth, usually within 30 minutes, using calibrated scales. Likewise gestational age is calculated from the crown rump length, measured at the dating scan between 9 and 14 weeks gestation. Given the pragmatic nature of the trial, non-routinely collected data items will be kept to a minimum. All sites will be provided with relevant database training"

Reviewer 2

4. Overstatement of the Causal Relationship Between Anaemia and Adverse Outcomes
Please see earlier.
We have revised the manuscript and the discussion section, see earlier.

5. "We have clarified our approach to data collection and addressed the matter of birth weight measurement by adding the following to the section Outcome measures and End points: To maximise the efficiency of the trial and reduce the burden on participating sites, routine, accurate and reproducible data will be extracted at source from the maternity and neonatal information systems and used for the analysis of the effectiveness of the intervention. For example the practice for measuring birth weight is to do this as soon as possible after birth, usually within 30 minutes, using calibrated scales. Likewise gestational age is calculated from the crown rump length, measured at the dating scan between 9 and 14 weeks gestation. Non-routinely collected data items will be kept to a minimum and all sites will be provided with relevant database training."

6. Inadequate Discussion of Potential Risks of Universal Supplementation
We have updated the discussion section to take account of this comment as also suggested by the editor– see above.

7. Discussion of the status of iron heterogeneity
We have addressed this point – please see response under major revisions

8. Justification of dose selection
We have addressed this point on the text and referenced our previous publication where full details of the pilot trial's results have been provided.

9. International recruitment
We have clarified this point – please see response under major revisions

10. Long term neurodevelopmental outcomes
We have addressed this point – please see response under major revisions

11. Reliance on Behavioural adherence support
This has been addressed and our response found under major revisions.

12. Recruitment bias
This has been addressed and our response found under major revisions.

Reviewer 3

1. Editorial review for syntax – we have checked the manuscript and updated the text and hope that it now meets the standards required.

2. Ethical consideration – we have added a section on ethical considerations and dissemination after the section on economic evaluation.

3. Start date for enrolment
   The trial opened on 23rd October 2024 and the first participant was recruited on 29th October 2024.
   We have added to the paper

4. Inclusion exclusion criteria – we have made clear our reference to these in table 1 in the text.

5. Development of anaemia – we have added the following passage for clarification –
   No additional blood sampling will be undertaken for the trial. Data on haematological indices will be taken from the routinely taken samples at first registration of the pregnancy in the first trimester and then at 28 weeks gestation. Data will also be collected on routine haematological indices at the time of making a diagnosis of anaemia, if that is outside of the routine screening time points

6. Target and recruits only in the first trimester a limitation for late bookers. – we have added a sentence in the discussion to highlight this problem although we think it will be small.
