# Supplementary material for: Primary prevention of maternal anaemia to prevent preterm delivery and other adverse outcomes (PANDA): a protocol for a double-blind placebo-controlled randomised trial of a daily iron supplement during pregnancy
Source: BMJ Open. 2026 Sep 28;16(9):e122579. doi: 10.1136/bmjopen-2026-122579 (PMC13629978; doi:10.1136/bmjopen-2026-122579)
Supplement: Supplementary file 2 [file bmjopen-16-9-s001.docx]

**APPENDIX**

**Process evaluation**

Strategies for adherence were developed as part of an initial workstream as part of a funded NIHR PGfAR grant.^22^ Adherence to the intervention is encouraged in two ways for the main trial. First, through the provision of an adherence intervention at recruitment, and throughout the trial via text messages [1/week] and website engagement. The intervention was co-designed with key stakeholders (service users, healthcare professionals, researchers), following formative research involving qualitative interviews with women (with and without a diagnosis of maternal anaemia), and healthcare professionals (e.g. midwives, obstetricians) to assess attitudes towards oral iron for the prevention of anaemia in pregnancy, and barriers and enablers to the uptake and adherence to oral iron supplementation. The intervention also draws on behaviour change theories and frameworks, including the Theoretical Domains Framework,^47^ Leventhal’s extended Common Sense Model,^48^ the Necessity Concerns Framework^49^ and the Behaviour Change Wheel.^23^ The intervention was piloted in an earlier trial, and refined following qualitative feedback from women and midwives on the acceptability and feasibility of the intervention. The resulting intervention delivered as part of the definitive trial described in this protocol includes a range of behaviour change techniques^50^ aiming to support the participant to take iron supplements and ongoing subsequent adherence. The specific intervention activities are described in Table 3. It will be delivered to all participants recruited into the trial irrespective of the trial arm they are randomised to.

All other care delivered to participants will follow local and national guidelines,^51^ and be delivered by maternity healthcare professionals as per standard care for all pregnant women. No other changes will be made to their pregnancy care plan.

Methods to assess each outcome are described in turn below:

***Fidelity***

The fidelity assessment aims to assess the extent to which the interventions are delivered and engaged with as intended. Guided by the National Institute of Health Behaviour Change Consortium Fidelity framework,^52^ we will assess fidelity across multiple domains of training, delivery, receipt and enactment. For fidelity of training and delivery, the trial includes fidelity enhancement strategies, including standardised training for research midwives and site initiation visits, intervention manuals, and delivery checklists to prompt delivery of all intervention components. The checklists will also enable gathering of provider self-reported fidelity, whereby for all recruited participants to the trial, research midwives delivering the intervention will be asked to rate the extent to which each intended intervention component was fully/partially/or not at all delivered, with free text responses to capture any reasons for not delivering the intervention as planned and any adaptations. The extent to which participants recall receiving the intervention and engaging with it will be explored in interviews and surveys described below, as well as via web analytics of the PANDA website.

***Feasibility and acceptability***

A mixed-methods approach will combine in-depth qualitative interviews with a sub-sample of participants (pregnant women and midwives) and quantitative surveys (with pregnant women) in up to the full trial sample, to explore participants’ attitudes towards the intervention and factors influencing adherence and engagement. The interview guide and survey items will be informed by the domains of the Theoretical Framework of Acceptability^53^ and the Capability, Opportunity and Motivation Model of behaviour (COM-B).^23^

***Interviews***

We will use a maximum variation sampling strategy, aiming for representation across sites, and demographic groups (e.g. age, ethnicities, geographical location) to recruit 30 pregnant women for interviews; sample size contingent on whether thematic saturation is reached.^54^ Women on the acute treatment arm will be recruited at week 6+1 postnatal/postpartum. Unblinding of womens’ study arm, for the purpose of recruitment for interviews, will be undertaken as outlined above under the section Process Evaluation Unblinding. Semi-structured interviews (lasting 30-60 minutes) will explore womens’ understanding and perceived acceptability of oral iron and adherence intervention, plus reported barriers/enablers to initiating iron supplementation and on-going adherence. We will also carry out semi-structured interviews (lasting 30-60 minutes) with midwives who have delivered the intervention (at least one midwife per trial site (n=20)), to explore intervention acceptability and to understand any barriers or enablers to delivering the intervention with fidelity.

***Surveys***

The survey will be administered at the 28-week antenatal visit (alongside other data collection questionnaires at this timepoint) to all women participating in the trial, taking approximately 15 minutes to complete. The survey will contain items taking the form of belief statements corresponding to each of the domains from the COM-B^23^ and each of the domains from the Theoretical Framework of Acceptability.^53^ This will enable exploration of the barriers/enablers to adherence and the perceived intervention acceptability. Participants will rate their agreement on a 5-point Likert type scale.

Findings from the process evaluation will aid interpretation of observed trial outcomes and inform the development of an implementation strategy if the oral iron supplementation strategy is found to be effective and is thus rolled out and/or implemented on a larger scale. Alternatively, it may also inform intervention refinement and optimisation if implementation and adherence are found to be sub-optimal.
